# Genetic spectrum and clinical features of adult leukoencephalopathies in a Chinese cohort

**DOI:** 10.1002/acn3.51794

**Published:** 2023-05-26

**Authors:** Minglei Liu, Yangyang Wang, Changhe Shi, Yanpeng Yuan, Lanjun Li, Xiaoyun Zhang, Yuming Xu, Jing Yang

**Affiliations:** ^1^ Department of Neurology The First Affiliated Hospital of Zhengzhou University Zhengzhou Henan China; ^2^ NHC Key Laboratory of Prevention and treatment of Cerebrovascular Disease Zhengzhou Henan China; ^3^ Institute of Neuroscience Zhengzhou University Zhengzhou Henan China; ^4^ Henan Key Laboratory of Cerebrovascular Diseases Zhengzhou University Zhengzhou Henan China

## Abstract

**Objective:**

Leukoencephalopathies are a group of heterogeneous disorders characterized by the degeneration of white matter, resulting in a variety of progressive neurological symptoms. To date, over 60 genes linked to genetic leukoencephalopathies have been discovered through whole‐exome sequencing (WES) and long‐read sequencing. Nonetheless, the genetic diversity and clinical variability of these disorders among various racial groups remain largely unknown. Therefore, this study aims to analyze the genetic spectrum and clinical features of Chinese adult leukoencephalopathies and compare the genetic profiles in different populations.

**Methods:**

A total of 129 patients suspected of possible genetic leukoencephalopathy were enrolled and underwent WES and dynamic mutation analysis. Bioinformatics tools were used to predict the pathogenicity of these mutations. Skin biopsies were conducted for further diagnosis. Genetic data sources from different populations were collected from published articles.

**Results:**

Genetic diagnosis was established in 48.1% of patients, with WES identifying 57 pathogenic or likely pathogenic variants in 39.5% of cases. *NOTCH3* and *NOTCH2NLC* were the most common mutated genes, accounting for 12.4% and 8.5% of cases, respectively. Dynamic mutation analysis revealed *NOTCH2NLC* GGC repeat expansions in 8.5% of patients. Different mutations resulted in varying clinical symptoms and imaging findings. Comparisons of genetic profiles between different populations showed distinct mutational spectrums in adult leukoencephalopathies.

**Interpretation:**

This study highlights the importance of genetic testing for accurate diagnosis and improved clinical management of these disorders. It also sheds light on the genetic heterogeneity of adult leukoencephalopathies across different races, emphasizing the need for further research on this topic.

## Introduction

Leukodystrophies and genetic leukoencephalopathies are considered a rare group of degenerative white matter disorders leading to a progressive syndrome including various combinations of cognitive and neuropsychiatric changes, movement disorders, spasticity and seizures, accompanied by widespread cranial white matter change on MRI T2/fluid‐attenuated inversion recovery (FLAIR) sequences.^1^, ^2^ Given variable and overlapping presentations and similar imaging changes, it is not easy to delineate the subtypes of adult leukoencephalopathies only from clinical viewpoints and metabolic or biochemical testing. Meanwhile, patients with genetic leukoencephalopathies often endure a long miserable time before accepting a definitive diagnosis or may not receive a diagnosis at all.^3^ Leukodystrophies and genetic leukoencephalopathies can present in children or adults, advances have been made in the diagnostic approach in children, but little is made about genetic methods in adults.^4^ Therefore, genetic investigations are essential to make a definitive diagnosis of such disorders.

Along with the use of whole‐exome sequencing (WES) and analysis of phenotype–genotype correlation, more than 60 genes have been found to be associated with adult leukoencephalopathies representative of great genetic heterogeneity.^5^ However, given that the causative mutations were only ascertained in 13.3%–26% of affected patients with different ethnic origins using WES or focused exome sequencing, it remained difficult and elusive for genetic diagnosis of adult leukoencephalopathies.^5^, ^6^ Recently, noncoding GGC repeat expansions in *NOTCH2NLC*, *FMR1*, *NUTM2B‐AS1* have been identified as the causative dynamic mutations based on the long read sequencing technology for three types of adult leukoencephalopathies: neuronal intranuclear inclusion disease (NIID), fragile X tremor/ataxia syndrome (FXTAS) and oculopharyngeal myopathy with leukoencephalopathy (OPML), respectively.^7^ Notably, *NOTCH2NLC* GGC repeat expansions causing neuronal intranuclear inclusion disease has been regarded as the most common reason for adult‐onset nonvascular leukoencephalopathies in Japanese populations, while this disorder is not common in European populations with adult‐onset leukoencephalopathy.^8^, ^9^ Therefore, it is necessary to further conduct genetic researches on adult leukoencephalopathies by combining WES and dynamic mutation analysis, especially in different populations.

In this study, we conducted WES and dynamic mutation analysis on a cohort of Chinese patients diagnosed with adult leukoencephalopathies. Subsequently, we performed genotype–phenotype correlation analysis to enhance the clinical recognition of these disorders. Furthermore, we compared the genetic profiles of this cohort with those of other populations, in order to elucidate the ethnic variation in the manifestation of adult leukoencephalopathies.

## Materials and Methods

### Patients

Patients were widely recruited from the Department of Neurology in the First Affiliated Hospital of Zhengzhou University between June 2019 and May 2022. The primary inclusion criteria comprised of (1) the observation of prominent white matter hyperintensity (WMH) on T2/FLAIR MRI, (2) the manifestation of a progressive neurological syndrome, and (3) a minimum age of 16 years. Subjects were excluded if laboratory tests indicated the presence of acquired white matter disease caused by factors such as vascular, inflammatory, infective, neoplastic, toxic, or drug‐induced factors. A total of 129 patients, suspected of having possible genetic leukoencephalopathy, were ultimately enrolled in the study. All patients underwent comprehensive clinical evaluation, including standardized medical history and neurological examinations conducted by two senior neurologists. The study was approved by the Ethics Committee of the First Affiliated Hospital of Zhengzhou University, and all subjects provided informed consent.

### Whole‐exome sequencing

Genomic DNA was extracted from the peripheral blood leukocytes using the QIAamp DNA Blood Maxi Kit (QIAGEN). Whole‐exome sequencing (WES) was performed on Agilent Sure Select Human All Exon V6 products for capturing exons and Illumina HiSeq X Ten platform for sequencing. The frequency of our identified rare variants in healthy people was assessed by using the Genome Aggregation Database (gnomAD, http://www.gnomad‐sg.org/), Exome Aggregation Consortium (ExAC, http://exac.broadinstitute.org/), 1000 Genomes Project (http://www.1000genomes.org), and dbSNP (https://www. ncbi.nlm.nih.gov/projects/SNP/) databases. These variants with minor allele frequency (MAF) > 0.01 were removed. Copy number variations (CNVs) of disease‐causing genes were evaluated using a mean normalized coverage.

The effect of variants on gene function was evaluated based on missense variant predicted tools, including SIFT (http://sift.jcvi.org/), Polyphen‐2 (http://genetics.bwh.harvard.edu/pph2/), and CADD (http://cadd.gs.washington.edu/snv). All variants were classified as pathogenic, likely pathogenic, benign, likely benign, or uncertain significance according to the American College of Medical Genetics and Genomics (ACMG) guidelines.^10^ Only pathogenic and likely pathogenic variants were considered as the disease causative variants and the pathogenicity of the remaining variants needs further investigation. Possible disease causative variants were confirmed by Sanger sequencing on ABI 3730 XL Genetic Analyzer. Co‐segregation analysis was performed on all available family members. The novelty of causative variants was determined according to ClinVar (http://www.ncbi.nlm.nih.gov/clinvar) and HGMD (http://www.hgmd.org) databases.

### Dynamic mutation analysis

Repeat‐primed PCR (RP‐PCR) and fluorescence amplicon length PCR (AL‐PCR) were used to detect the presence of dynamic mutation and determine the exact trinucleotide repeat number in *NOTCH2NLC*, *FMR1*, and *NUTM2B‐AS1*, as described previously.^11^ Capillary electrophoresis of the PCR products was used to perform on A 3500× L Genetic Analyzer (Applied Biosystems) to analyze the fragment length, and the allele size was determined using GeneScan 1,000 ROX Size Standard (Applied Biosystems). Briefly, a saw‐tooth tail pattern in RP‐PCR electropherograms suggested the presence of trinucleotide repeat expansions, and the length of the highest signal peak in AL‐PCR electropherograms was used for fragment length. According to previous reports, *NOTCH2NLC* GGC repeat expansion has three forms: normal (<40 repeats), intermediate (41–60 repeats), and pathogenic (>60 repeats); and *FMR1* GGC repeat has four forms: normal (6–44 repeats), intermediate (45–54 repeats), premutation (55–200 repeats), and full mutation (>200 repeats).^12^, ^13^ Abnormal *NUTM2B‐AS1* CGG repeat expansion will be considered if the number of repeats is much higher than that of healthy controls according to our previous report.^14^

### Skin biopsy and immunohistochemistry

Three‐millimeter diameter skin punch biopsies were performed at the lateral distal leg and neck under 20 mg/ml lidocaine local anesthesia according to the previously reported protocol.^15^ The tissues were embedded in paraffin and sectioned at 4‐μm thickness using the Leica clinical microtome (HistoCore BIOCUT, Germany). The sections were stained with hematoxylin and eosin, anti‐ubiquitin antibody (ab7780, Abcam, 1:400), or anti‐p62 antibody (ab56416, Abcam, 1:800). Photomicrographs were captured with a fluorescence microscope (Nikon, Eclipse Ni).

### Genetic data from different populations

The genetic data for patients with adult leukoencephalopathies from various populations were obtained through a comprehensive search of published literature. The search was conducted on PubMed, using search terms such as “adult leukoencephalopathy” or “genetic leukoencephalopathy,” in combination with “whole‐exome sequencing” or “focus‐exome sequencing” or “*NOTCH2NLC*” or “*FMR1*” or “*NUTM2B‐AS1*.” A total of six articles, published between 2015 and 2022, containing detailed genetic data of Asians and Caucasians, were identified and reviewed. Additionally, reference lists of these articles were examined to locate further relevant literature for consultation.

## Results

### Genetic outcomes

**Figure 1:**
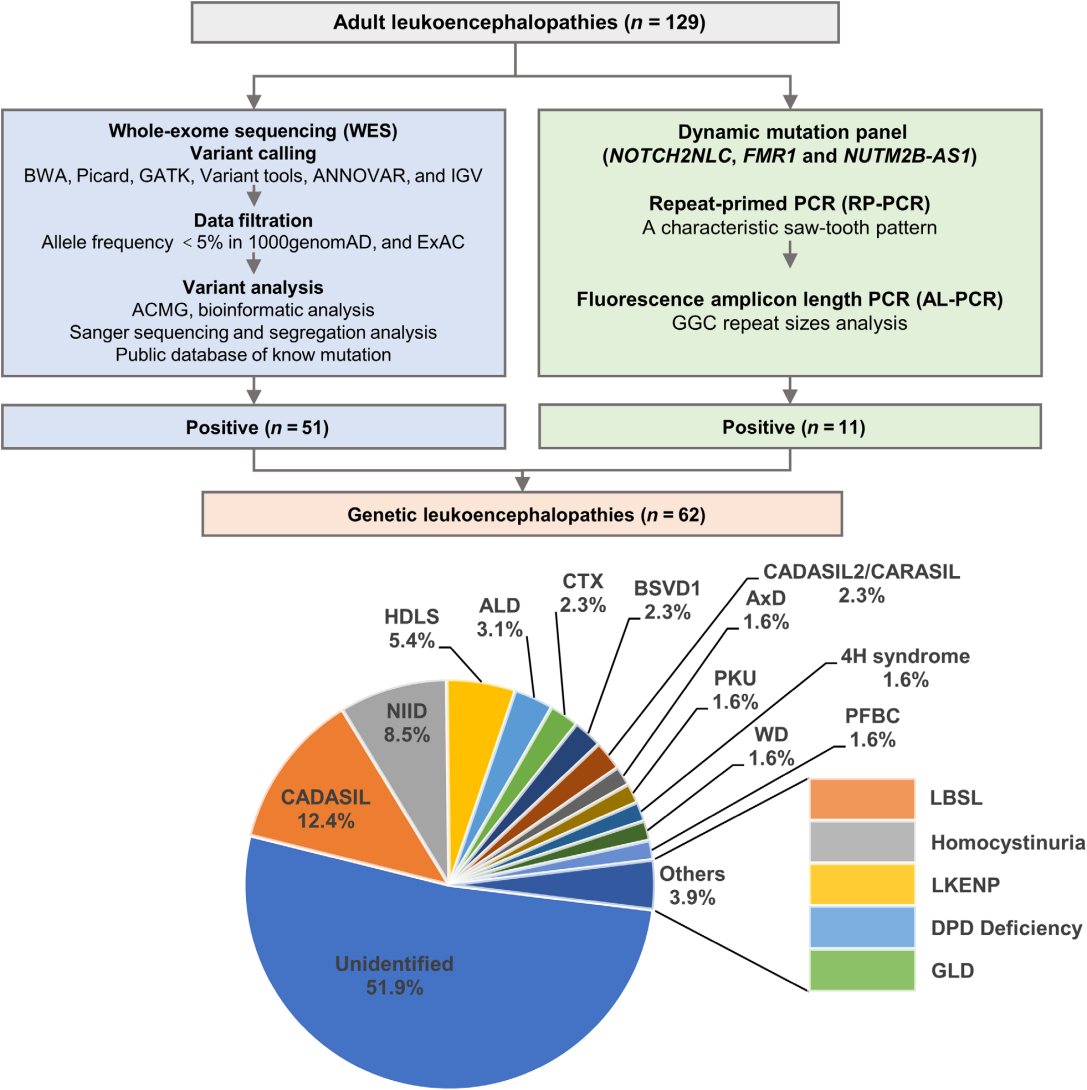
Genetic diagnostic flowchart and results in adult‐leukoencephalopathy patients. CADASIL, cerebral autosomal dominant arteriopathy with subcortical infarcts and leukoencephalopathy; NIID, neuronal intranuclear inclusion disease; HDLS, hereditary diffuse leukodystrophy with axonal spheroids; ALD, X‐linked adrenoleukodystrophy; CTX, Cerebrotendinous Xanthomatosis; BSVD1, brain small vessel disease with or without ocular anomalies; CARASIL, cerebral autosomal recessive arteriopathy with subcortical infarcts and leukoencephalopathy; AxD, Alexander disease; PKU, phenylketonuria; WD, Wilson disease; PFBC, primary familial brain calcification; LBSL, leukoencephalopathy with brainstem and spinal cord involvement and lactate elevation; LKENP, progressive leukodystrophy with ovarian failure; DPD, dihydropyrimidine dehydrogenase; GLD, globoid cell leukodystrophy, also known as Krabbe disease.

The flowchart and result of genetic analysis were shown in Figure 1. In detail, WES analysis identified 57 pathogenic or likely pathogenic variants including 22 novel variants (Table 1) and 35 known variants (Table S1) in 51 out of 129 adult leukoencephalopathy cases (39.5%). *NOTCH3*, causing cerebral autosomal dominant arteriopathy with subcortical infarcts and leukoencephalopathy (CADASIL),^16^ was the most common causative gene in our cohort (16/129, 12.4%), followed by *CSF1R*, causing hereditary diffuse leukodystrophy with axonal spheroids (HDLS, 7/129, 5.4%),^17^ *ABCD1*, causing X‐linked adrenoleukodystrophy (ALD, 4/129, 3.1%),^18^ *CYP27A1*, causing Cerebrotendinous Xanthomatosis (CTX, 3/129, 2.3%),^19^ *COL4A1*, causing brain small vessel disease with or without ocular anomalies (BSVD1, 3/129, 2.3%),^20^ *HTRA1*, causing CADASIL2 or cerebral autosomal recessive arteriopathy with subcortical infarcts and leukoencephalopathy (CARASIL, 3/129, 2.3%),^21^ *GFAP*, causing Alexander disease (AxD, 2/129, 1.6%),^22^ *PAH*, causing phenylketonuria (PKU, 2/129, 1.6%),^23^ *POLR3A* and *POLR3B*, causing 4H syndrome (2/129, 1.6%),^24^ *ATP7B*, causing Wilson disease (WD, 2/129, 1.6%),^25^ *JAM2* and *PDGFB*, causing primary familial brain calcification (PFBC, 2/129, 1.6%),^26^, ^27^ while *AARS2*, causing progressive leukodystrophy with ovarian failure (LKENP),^28^ *DARS2*, causing leukoencephalopathy with brainstem and spinal cord involvement and lactate elevation (LBSL),^29^ *CBS*, causing hereditary homocystinurias, *DPYD*, causing dihydropyrimidine dehydrogenase deficiency (DPD deficiency),^30^ and *GALC*, causing globoid cell leukodystrophy (GLD; also known as Krabbe disease),^31^ were relatively rare and only found in one case (1/129, 0.8%), respectively. Meanwhile, we detected variants of uncertain significance in *NOTCH3*, *HTRA1*, *COL4A1*, *APP*, *ABCD1*, *ATN1*, *CSF1R*, and *GFAP* in 16 patients (Table S2). However, no pathological CNVs were identified.

**Table 1:** Novel pathogenic/likely pathogenic variants identified in our cohort.

Gene	Nucleotide change	Amino acid change	Inheritance pattern	gnomAD	ExAC	SIFT	Polyphen‐2	CADD	ACMG
*NOTCH3*	c.269G > A	p.R90H	AD	4.54E‐05	5.88E‐05	D	Pro_D	D	LP (PM1 + PM2 + PM5 + PP2 + PP3 + PP4)
*NOTCH3*	c.709G > A	p.V237M	AD	0.0002	0.0002	D	Pro_D	D	LP (PM2 + PP1 + PP2 + PP3 + PP4)
*NOTCH3*	c.985 T > C	p.C329R	AD	—	—	D	Pro_D	D	LP (PM1 + PM2 + PM5 + PP2 + PP3 + PP4)
*NOTCH3*	c.1063 T > C	p.C355R	AD	—	—	D	Pro_D	D	LP (PM2 + PP1 + PP2 + PP3 + PP4)
*NOTCH3*	c.1649C > T	p.S550F	AD	8.14E‐06	—	T	Pro_D	D	LP (PM1 + PM2 + PP2 + PP3)
*NOTCH3*	c.3172G > T	p.G1058C	AD	—	—	D	Pro_D	D	LP (PM1 + PM2 + PP3 + PP4)
*NOTCH3*	c.5467G > A	p.D1823N	AD	2.03E‐05	2.47E‐05	D	Pro_D	D	LP (PM2 + PP1 + PP2 + PP3 + PP4)
*CSF1R*	c.1736G > A	p.R579Q	AD	—	—	D	Pro_D	D	LP (PM1 + PM2 + PP3 + PP4)
*CSF1R*	c.1949 T > C	p.L650P	AD	—	—	D	Pro_D	D	LP (PM1 + PM2 + PP2 + PP3)
*CSF1R*	c.2570C > T	p.P857L	AD	—	—	D	Pro_D	D	LP (PM1 + PM2 + PM5 + PP3)
*CYP27A1*	c.1004C > T	p.A335V	AR	—	—	D	Pro_D	D	LP (PS1 + PM2 + PP3)
*COL4A1*	E16‐17 del	‐	AD	—	—	NA	NA	NA	LP (PS2 + PM2 + PP4)
*HTRA1*	c.62C > A	p.S21*	AD	—	—	NA	NA	D	LP (PM2 + PM4 + PP3 + PP4)
*HTRA1*	c.176G > C	p.R59P	AR	—	—	T	B	D	VUS (PM2 + PP4)
*HTRA1*	c.971A > G	p.N324S	AD	—	—	D	Pro_D	D	LP (PM1 + PM2 + PP3 + PP4)
*GFAP*	c.263G > A	p.R88H	AD	2.44E‐05	3.30E‐05	T	Pro_D	D	LP (PM1 + PM2 + PM5 + PP3)
*POLR3B*	c.361C > T	p.R121*	AR	4.06E‐06	8.24E‐06	NA	NA	D	LP (PM2 + PM4 + PP3 + PP4)
*POLR3B*	c.2698C > T	p.R900C	AR	4.07E‐06	8.34E‐06	D	Pro_D	D	VUS (PM2 + PP3 + PP4)
*JAM2*	c.460C > T	p.R154*	AR	2.03E‐05	1.66E‐05	NA	NA	D	LP (PM2 + PM3 + PM4 + PP3 + PP4)
*PDGFB*	c.148G > T	p.G50*	AD	—	—	NA	NA	D	LP (PM2 + PM4 + PP1 + PP3 + PP4)
*AARS2*	c.2557C > T	p.R853W	AR	4.07E‐06	8.38E‐06	D	Pro_D	D	LP (PM2 + PM3 + PP3 + PP4)
*DPYD*	c.751C > T	p.L251F	AR	—	—	D	Pro_D	D	LP (PM1 + PM2 + PM3 + PP3)

gnomAD, Genome Aggregation Database; ExAC, Exome Aggregation Consortium; ACMG, American College of Medical Genetics and Genomics; AD, autosomal dominant; AR, autosomal recessive; D, damaging; Pro_D, probably damaging; NA, not available; LP, likely pathogenic; VUS, variants of uncertain significance.

To assess the unresolved genetic causes in our cohort, we subsequently investigated trinucleotide repeat expansions of 3 additional genes (*NOTCH2NLC*, *FMR1*, and *NUTM2B‐AS1*) in the 129 patients. Surprisingly, we found *NOTCH2NLC* GGC expansions in 11 patients, which was the second most common causative gene in our cohort (11/129, 8.5%). The number of repeats of *NOTCH2NLC* GGC is 66 to 170, which can be used as a diagnostic basis for NIID. However, no patients harbored expanded trinucleotide repeats of *FMR1* or *NUTM2B‐AS1*.

### Clinical characteristics

Among the 62 patients with genetic leukoencephalopathies, 38 were male and 24 were female. The average age at onset was 39.7 ± 15.7 (range 7–76) years, and the age of diagnosis was 43.3 ± 14.8 (range 18–76) years. Thirty (48.4%) patients had a positive family history. Detailed clinical features and imaging findings were shown in Table 2; Tables S3 and S4.

**Table 2:** Clinical features and imaging findings in the patients with genetic leukoencephalopathies.

Diagnosis	Gene	Number of cases	Sex ratio (M/F)	Average age	Average AAO	Familial history	Common clinical symptoms	Imaging findings
Leukoencephalopathies with *NOTCH2NLC* GGC repeat expansion								
NIID	*NOTCH2NLC*	11	5/6	59.9	57.3	3	Cognitive decline (8), autonomic dysfunction (5), limb weakness (4), headache (4), parkinsonism (3)	Symmetrical and confluent WMHs, mostly in the frontoparietal lobes (11), deep white matter and periventricular area involving the U‐fibers (10), high signals along with the corticomedullary junction on DWI (9)
Inherited vascular leukoencephalopathies								
CADASIL	*NOTCH3*	16	10/6	44.7	42.2	15	Stroke/TIA (11), cognitive decline (5), dizziness (5), migraine (4), ataxia (4)	Multifocal WMHs in the white matter (16), especially in the anterior temporal lobe (15) and external capsule (11), multiple lacunar infarcts (9) and cerebral microbleeds (3)
CARASIL/CADASIL2	*HTRA1*	3	2/1	52.0	49.0	2	Cognitive decline (3), stroke (2), parkinsonism (1), spastic gait (1)	Similar to CADASIL. Less involvement of anterior temporal lobe (1) and external capsule (1)
BSVD1	*COL4A1*	3	2/1	40.7	39.3	2	Ataxia (3), stroke (2), cognitive decline (1), parkinsonism (1)	Prominent WMHs in the frontoparietal (3) and periventricular area (3), multiple lacunar infarcts (3) and microbleeds (2)
Inherited metabolic disorders with leukodystrophies								
ALD	*ABCD1*	4	4/0	44.0	39.0	1	Peripheral neuropathy (3), spastic gait (3), cognitive decline (2), autonomic dysfunction (2)	Frontoparietal and paraventricular pattern of white matter involvement (4), DWI abnormality (3)
CTX	*CYP27A1*	3	2/1	25.7	18.3	0	Ataxia (2), cataract (2), muscle atrophy (1), peripheral neuropathy (1), epilepsy (1)	WMHs in the deep periventricular white matter (3), brainstem (1) and cerebellum (1)
PKU	*PAH*	2	2/0	19.5	17.0	0	Psychological disorders (2), headache (1), cognitive decline (1), ataxia (1)	Symmetric periventricular (2) and lobar (1) white matter involvement
Homocystinurias	*CBS*	1	1/0	53.0	41.0	0	Limb numbness, weakness	Symmetric WMHs in the frontoparietal lobes and paraventricular area
DPD deficiency	*DPYD*	1	1/0	33.0	33.0	0	Cognitive decline, blurred vision	Symmetric WMHs in the posterior horn of the lateral ventricle, with restricted diffusion on DWI
Krabbe disease	*GALC*	1	1/0	27.0	17.0	0	Epilepsy, diplopia, blurred vision	WMHs in the right frontal lobe
Inherited CNS demyelinating leukodystrophies								
HDLS	*CSF1R*	7	3/4	40.4	39.3	5	Cognitive decline (5), dysarthria (4), ataxia (4), parkinsonism (3), peripheral neuropathy (3)	Frontal and paraventricular pattern of white matter involvement, with restricted diffusion on DWI (7)
AxD	*GFAP*	2	1/1	52.5	49.0	2	Parkinsonism (1), limb weakness (1), autonomic dysfunction (1), ataxia (1)	Symmetric WMHs in frontoparietal lobes and paraventricular area (2), with medulla atrophy (1)
LKENP	*AARS2*	1	1/0	19.0	19.0	0	Ataxia, headache, tremor	Bilateral frontoparietal lobes, periventricular area, and corpus callosum white matter involvement
LBSL	*DARS2*	1	1/0	18.0	18.0	0	Ataxia, peripheral neuropathy	Bilateral centrum semiovale, paraventricular area, cerebellum, and brainstem whiter matter involvement
Inherited CNS hypomyelinating leukodystrophies								
4H syndrome	*POLR3A/B*	2	0/2	40.5	18.0	0	Ataxia (2), cognitive decline (1), epilepsy (1), limb weakness (1)	Diffuse WMHs in the cerebral white matter, hypomyelination (2)
Other diseases with white matter involvement								
WD	*ATP7B*	2	1/1	35.5	28.0	0	Dysarthria (2), postural tremor (2), dysphagia (1)	Symmetric WMHs in the frontoparietal lobe, external capsule and brainstem (2)
PFBC	*JAM2/PDGFB*	2	2/0	25.0	24.5	0	Ataxia (1), dizziness (1)	Symmetric WMHs in the lobar and periventricular area (2)

M, male; F, female; CADASIL, cerebral autosomal dominant arteriopathy with subcortical infarcts and leukoencephalopathy; TIA, transient ischemic attack; WMH, white matter hyperintensity; CARASIL, cerebral autosomal recessive arteriopathy with subcortical infarcts and leukoencephalopathy; BSVD1, brain small vessel disease with or without ocular anomalies; ALD, X‐linked adrenoleukodystrophy; CTX, Cerebrotendinous Xanthomatosis; PKU, phenylketonuria; DPD, dihydropyrimidine dehydrogenase; DWI, diffusion weighted imaging; HDLS, hereditary diffuse leukodystrophy with axonal spheroids; AxD, Alexander disease; LKENP, progressive leukodystrophy with ovarian failure; LBSL, leukoencephalopathy with brainstem and spinal cord involvement and lactate elevation; WD, Wilson disease; PFBC, primary familial brain calcification; NIID, neuronal intranuclear inclusion disease.

### Leukoencephalopathies with *NOTCH2NLC* GGC repeat expansion

**Figure 2:**
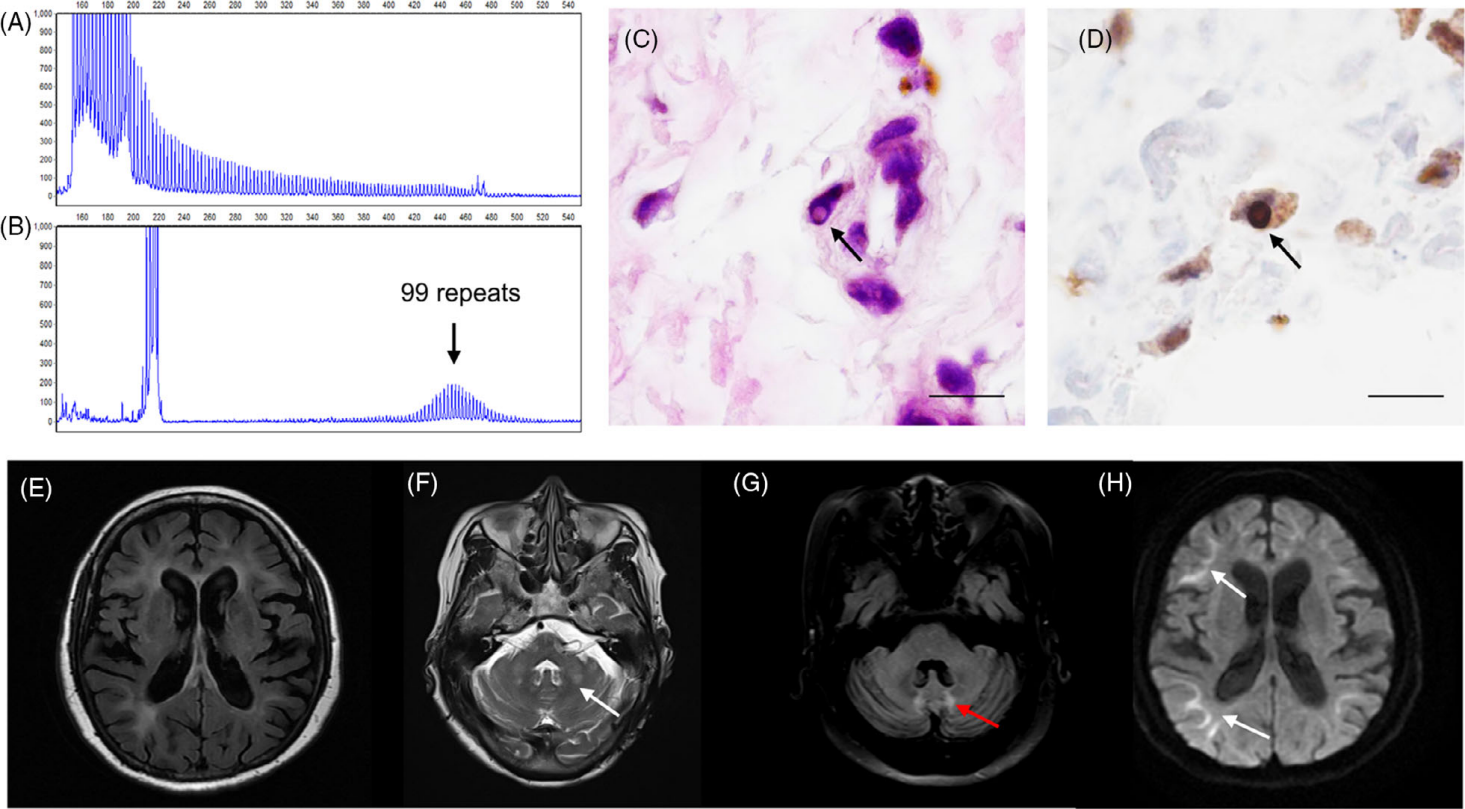
Representative gene testing results, skin pathological features, and MRI findings of NIID patients. (A) Repeat‐primed polymerase chain reaction (RP‐PCR) shows a characteristic saw‐tooth pattern, suggesting an abnormal GGC expansion in the *NOTCH2NLC* gene. (B) Fluorescence amplicon length PCR (AL‐PCR) indicates the patient has 99 GGC repeats in the *NOTCH2NLC* gene. (C) Hematoxylin and eosin (HE) staining shows representative eosinophilic intranuclear inclusions in the fibroblasts (arrow). (D) Immunohistochemical staining with anti‐ubiquitin antibodies shows representative ubiquitin‐positive intranuclear inclusions in the fibroblasts (arrow). (E) Axial FLAIR image shows white matter hyperintensities in the subcortical and periventricular white matter. (F) Axial T2‐weighted MRI image shows hyperintensities in the cerebellar middle peduncle (white arrow). (G) Axial FLAIR image shows hyperintensities in the cerebellar vermis (red arrow). (H) Axial DWI image shows representative hyperintensities along the corticomedullary junction. NIID, neuronal intranuclear inclusion disease; FLAIR, fluid‐attenuated inversion recovery; DWI, diffusion weighted imaging. Scale bar = 10 μm.

Eleven patients were diagnosed with neuronal intranuclear inclusion disease (NIID) as a result of abnormal *NOTCH2NLC* GGC repeats (Fig. 2A and B). Skin biopsies were performed on all patients and exhibited eosinophilic and ubiquitin‐positive intranuclear inclusions in the fibroblasts, sweat gland cells, and adipocytes (Fig. 2C and D).

Among the 11 NIID patients, three (27.3%) had a positive family history, and the mean age at onset was 57.3 ± 10.4 years (range: 46–76 years). The most common initial symptom was cognitive decline (4, 36.4%), followed by limb weakness (3, 27.3%). Headache and limb numbness were the initial symptoms of two (18.2%) and one (9.1%) patient(s), respectively. One patient initially experienced a transient ischemic attack (TIA) and subsequently developed cognitive decline, parkinsonism, and headache. During the disease progression, cognitive decline became the most prevalent symptom (8, 72.7%), followed by autonomic dysfunction (5, 45.5%), limb weakness (4, 36.4%), headache (4, 36.4%), parkinsonism (3, 27.3%), loss of consciousness (2, 18.2%), limb numbness (2, 18.2%), psychological disorders (2, 18.2%), and ataxia (2, 18.2%).

Brain MRI scans of the NIID patients revealed symmetrical and confluent white matter hyperintensities (WMHs) on T2/FLAIR, which were primarily distributed in the frontoparietal lobes (100.0%) and deep and periventricular areas, including the U‐fibers (90.9%; Fig. 2E). WMHs were also present in the corpus callosum (54.5%), brainstem (27.3%), temporal lobe (27.3%), and occipital lobe (18.2%). WMH in the cerebellar middle peduncle and vermis was observed in only one patient (Fig. 2F and G). Hyperintense signals were detected in nine patients (81.8%) on DWI, mainly in the frontoparietal lobes, along with the corticomedullary junction (Fig. 2H). Additional features, such as calcifications, infarcts, and microbleeds, were present in 4 (36.4%), 3 (27.3%), and 3 (27.3%) patients, respectively. Enhanced MRI scans were available in four patients, and none showed enhancement in the lesions.

### Inherited vascular leukoencephalopathies

**Figure 3:**
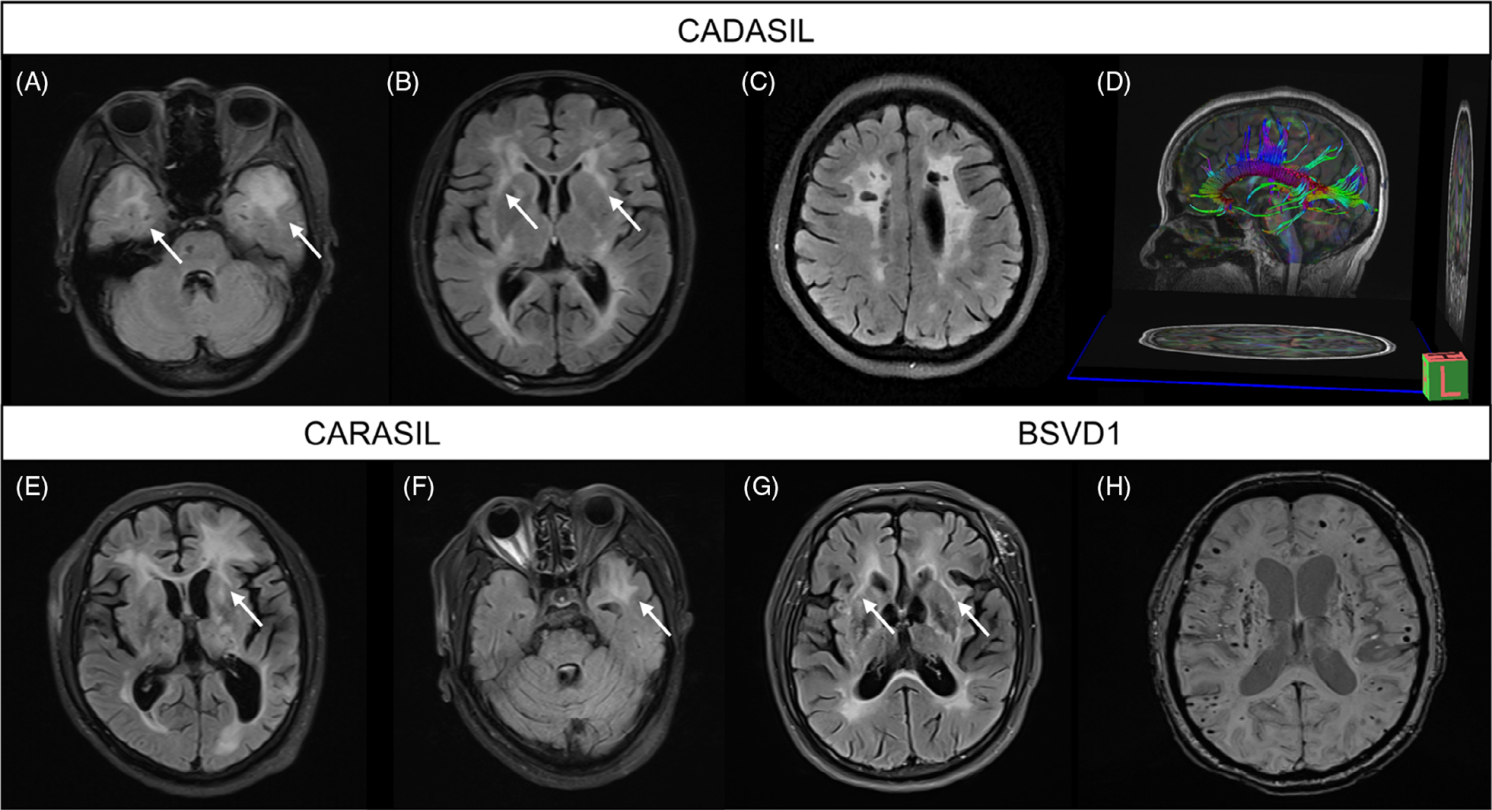
Representative MRI findings of patients with inherited vascular leukoencephalopathies. (A–D) Typical MRI findings of CADASIL patients. Axial FLAIR images show white matter lesions in the anterior temporal lobes (A), external capsules, and periventricular area (B), as well as multiple infarcts in the frontoparietal lobes (C). Sagittal DTI image shows obvious sparse and interrupted fibers in the corpus callosum bundle (D). (E and F) Typical MRI findings of CARASIL patients. Axial FLAIR images show asymmetrical white matter hyperintensities in the frontal lobe, basal ganglia, thalamus, external capsule, paraventricular area (E), as well as the left anterior temporal lobe (F). (G and H) Typical MRI findings of BSVD1 patients. Axial FLAIR image shows white matter hyperintensities in the periventricular area, internal and external capsules (G). Axial SWI image shows diffuse microbleeds in the frontoparietal lobes and basal ganglia (H). CADASIL, cerebral autosomal dominant arteriopathy with subcortical infarcts and leukoencephalopathy; FLAIR, fluid‐attenuated inversion recovery; DTI, diffusion tensor imaging; CARASIL, cerebral autosomal recessive arteriopathy with subcortical infarcts and leukoencephalopathy; BSVD1, brain small vessel disease with or without ocular anomalies; SWI, susceptibility weighted imaging.

The present study identified a total of 22 patients with mutations in *NOTCH3*, *HTRA1*, and *COL4A1*, which are known to cause cerebral small vessel disease (SVD). Among these, 16 patients with *NOTCH3* mutations had an average age at onset of 42.2 ± 13.8 years, with ischemic stroke or TIA being the most common clinical symptom (11, 68.8%). Other common symptoms included cognitive decline (5, 31.3%), dizziness (5, 31.3%), migraine (4, 25.0%), and ataxia (4, 25.0%). One patient with *NOTCH3* mutations presented with typical parkinsonism and had a poor response to levodopa treatment. Prominent WMHs on T2/FLAIR were observed in the frontoparietal lobe and periventricular area in all CADASIL patients, with almost all patients (15/16, 93.8%) presenting with WMHs in the temporal lobe, particularly in the temporal poles (Fig. 3A). In addition, WMH in the external capsule was a specific manifestation and was observed in 11 (68.8%) patients (Fig. 3B). Multiple lacunar infarcts (Fig. 3C) and cerebral microbleeds occurred in 9 (56.2%) and 3 (18.8%) patients, respectively. Diffusion tensor imaging (DTI) was available in one case and showed diffuse tract damage (Fig. 3D).

Furthermore, three patients with mutations in *HTRA1* were identified, two of whom carried single heterozygous mutations causing CADASIL2, and one patient carried compound heterozygous mutations causing CARASIL. The average age at onset was 49.0 ± 4.0 (range 45–53) years. Two CADASIL2 patients presented with symptoms such as ischemic stroke, cognitive decline, and psychiatric symptoms. The CARASIL patient presented with spastic gait, low back pain, cognitive decline, and parkinsonism. The image manifestations of CADASIL2 and CARASIL were similar to those of CADASIL, with prominent WMHs in the frontoparietal lobes and periventricular area, and multiple lacunar infarcts. WMH in the external capsule was observed in one CADASIL2 patient and one CARASIL patient (Fig. 3E), and typical temporal pole involvement was only found in the CARASIL patient (Fig. 3F). Additionally, a lumbar MRI revealed herniated disks with degenerative changes in one CADASIL2 patient.

Moreover, three patients with mutations in *COL4A1* were identified, with an average age at onset of 39.3 ± 3.1 years. Two patients presented with ischemic stroke, followed later by ataxia and limited eye movement. One patient started with parkinsonism and then developed ataxia and cognitive decline. MRI in these cases demonstrated prominent WMHs in the frontoparietal and periventricular area without the involvement of temporal poles, but the involvement of the external capsule was found in one case (Fig. 3G). Multiple lacunar infarcts were observed in all cases. Susceptibility weighted imaging (SWI) was available in two cases and revealed pontine and occipital microbleeds in one patient and diffuse microbleeds affecting lobar, thalamic, basal ganglia, and cerebellar regions in the other patient (Fig. 3H).

### Inherited metabolic disorders with leukodystrophies

**Figure 4:**
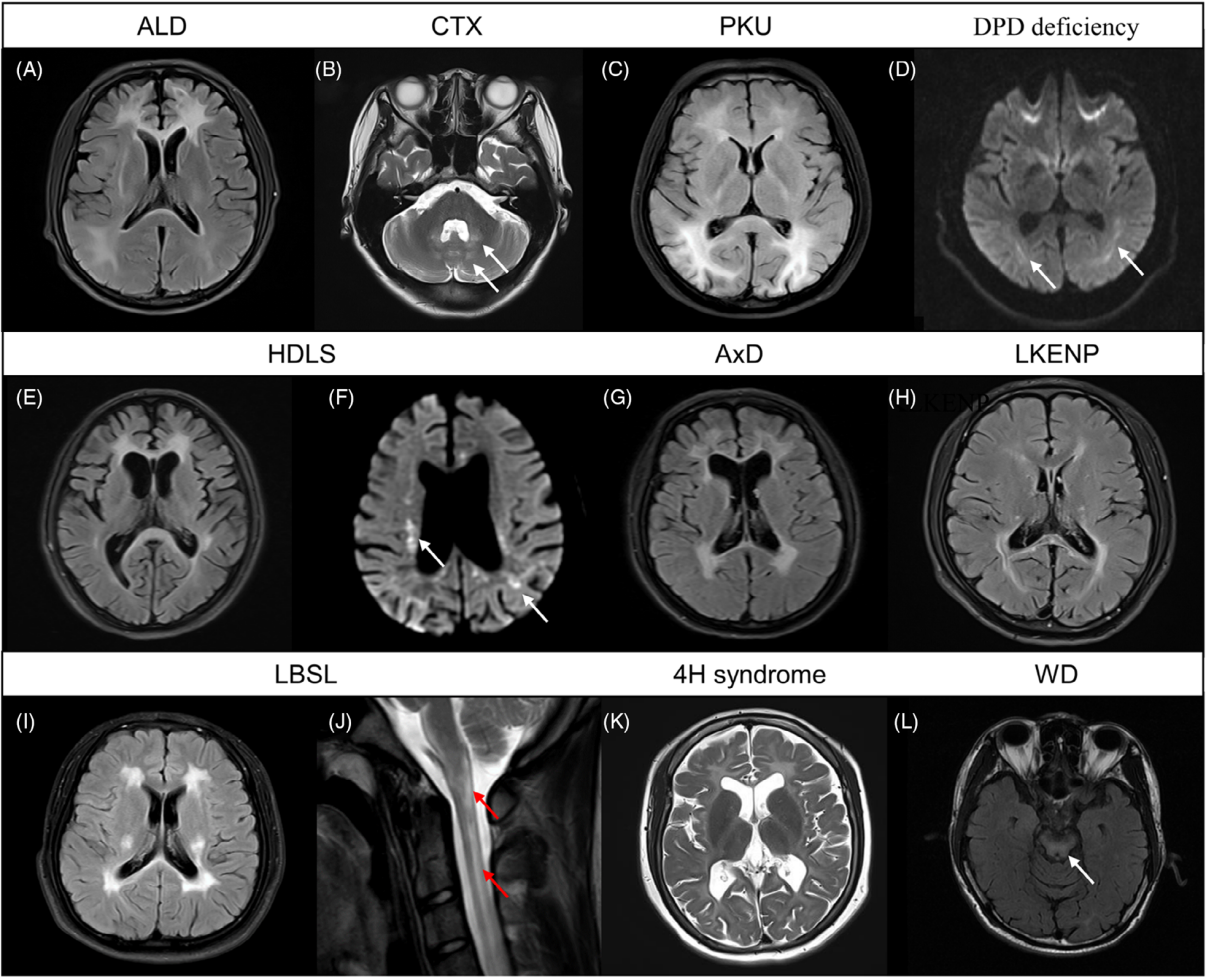
MRI findings of patients with inherited metabolic disorders, demyelinating leukodystrophies, hypomyelinating leukodystrophies, and other diseases with white matter involvement. (A) Axial FLAIR image shows frontal and parieto‐occipital white matter involvement in an ALD patient. (B) Axial T2‐weighted MRI image shows cerebellar white matter involvement in a CTX patient. (C) Axial FLAIR image of a PKU patient shows white matter hyperintensities in the frontal lobe, posterior horn of lateral ventricle extending to the cortex. (D) Axial DWI image of a DPD deficiency patient reveals restricted diffusion in the posterior horn of the lateral ventricle. (E) Axial FLAIR image of an HDLS patient shows white matter hyperintensities in the anterior horn of the lateral ventricle and splenium of the corpus callosum. (F) Axial DWI image of the HDLS patient shows scattered hyperintensities in the periventricular area and corticomedullary junction extending to the cortex. (G) Axial FLAIR image of an AxD patient shows hyperintensities in the anterior and posterior horn of the lateral ventricle. (H) Axial FLAIR image of the LKENP patient reveals hyperintensities in the posterior horn of the lateral ventricle and splenium of the corpus callosum. (I) Axial FLAIR image of the LBSL patient shows hyperintensities in the anterior and posterior horn of the lateral ventricle. (J) Sagittal T2‐weighted MRI image of this patient shows hyperintensities in the posterior columns of the spinal cord. (K) Axial T2‐weighted MRI image of a 4H syndrome shows diffuse hyperintensities in the lobes, external capsules, and periventricular area. (L) Axial FLAIR image of a WD patient shows hyperintensities in the midbrain. FLAIR, fluid‐attenuated inversion recovery; ALD, X‐linked adrenoleukodystrophy; CTX, Cerebrotendinous Xanthomatosis; PKU, phenylketonuria; DWI, diffusion weighted imaging; DPD, dihydropyrimidine dehydrogenase; HDLS, hereditary diffuse leukodystrophy with axonal spheroids; AxD, Alexander disease; LKENP, progressive leukodystrophy with ovarian failure; LBSL, leukoencephalopathy with brainstem and spinal cord involvement and lactate elevation; WD, Wilson disease.

The present study identified a cohort of patients who were diagnosed with leukodystrophies associated with inherited metabolic disorders resulting from mutations in genes related to inborn errors of metabolism. Specifically, four male patients were diagnosed with ALD due to carrying a hemizygote mutation in *ABCD1*, with an average age at onset of 39.0 ± 6.6 (range 31–47) years. The clinical symptoms included peripheral neuropathy (3, 75.0%), spastic gait (3, 75.0%), cognitive decline (2, 50.0%), autonomic dysfunction (2, 50.0%), dysarthria (1, 25.0%) and tremor (1, 25.0%). Brain MRI revealed that WMHs were concentrated in the frontoparietal lobes and paraventricular area, corpus callosum, and brainstem, with diffusion‐weighted imaging (DWI) abnormality and calcifications found in some cases. Additionally, subcortical U‐fibers and external capsule were affected in one case (Fig. 4A), and contrast enhancement showed slight enhancement of the lesion.

Furthermore, three patients carrying homozygous compound heterozygous *CYP27A1* mutations causing CTX exhibited clinical symptoms such as ataxia, cataract, muscle atrophy, peripheral neuropathy, epilepsy, and pescavu. Brain MRI showed WMHs in the periventricular area, brainstem, and cerebellum (Fig. 4B), with DWI abnormality found in one case.

Two patients with compound heterozygous *PAH* mutations causing PKU exhibited clinical symptoms of headache, cognitive decline, ataxia, and personality change, with WMHs found in the periventricular area, lobar area, and corpus callosum (Fig. 4C). After 1 year of treatment with a low phenylalanine diet, their clinical symptoms improved and the WMHs were significantly reduced.

Moreover, the study identified three patients with compound heterozygous *CBS* mutations causing homocystinurias, compound heterozygous *DPYD* mutations causing DPD deficiency, and homozygous *GALC* mutations causing Krabbe Disease, respectively. These patients exhibited clinical symptoms such as lower limb numbness and weakness, cognitive decline, blurred vision, and generalized epileptic seizure. Brain MRI revealed WMHs in different regions, with restricted diffusion found in some cases (Fig. 4D).

### Inherited CNS demyelinating leukodystrophies

The study identified patients with various mutations causing inherited CNS demyelinating leukodystrophies. Seven patients with *CSF1R* mutations causing HDLS were identified, with mean age at onset of 39.3 ± 7.6 (range 29–49) years. Dysarthria was the initial presentation in three patients (42.9%), while the remaining patients presented with parkinsonism, ataxia, dizziness, and cognitive decline. During the disease course, cognitive decline was the most common symptom (71.4%), followed by dysarthria (57.1%), ataxia (57.1%), parkinsonism (42.9%), peripheral neuropathy (42.9%), psychological disorders (28.6%), dizziness (28.6%), and autonomic dysfunction (28.6%). Parkinsonism‐like symptoms were not due to Parkinson's disease, as skin biopsies did not show phosphorylated‐α‐synuclein and L‐dopa treatment was not effective. MRI findings included frontoparietal lobar and periventricular WMHs (Fig. 4E) in all cases (100%), as well as WMHs in the corpus callosum (71.4%), temporal lobe (57.1%), brainstem (42.9%), and occipital lobe (28.6%). Brain calcifications were found in two cases (28.6%). Extra DWI signal abnormality was observed in the periventricular area and the corticomedullary junction extending to the cortex (Fig. 4F). Contrast‐enhanced MR and MR spectroscopy were available in one patient, revealing increased Cho concentrations indicating gliosis and slight enhancement at the lesions.

In addition, two patients with *GFAP* mutations causing AxD were identified. One patient presented with limb weakness at the age of 34, and over the next 4 years, she developed progressive bradykinesia and ataxia. Similar symptoms were observed in her sister, who carried the same mutation. MRI showed extensive WMHs in the frontoparietal lobes and periventricular area, as well as atrophy and signal intensity changes in the medulla. The other patient was a 67‐year‐old farmer with progressive parkinsonism and autonomic dysfunction for 3 years. MRI demonstrated symmetric WMHs in the frontoparietal lobes and paraventricular area (Fig. 4G), while the brainstem was not obviously involved.

Moreover, compound heterozygous mutations in *AARS2* and *DARS2* were detected in two male patients with slowly progressive ataxia and peripheral neuropathy. MRI findings in the former patient revealed extensive WMHs in the bilateral frontoparietal lobes, periventricular area, and corpus callosum (Fig. 4H). The latter patient showed diffuse WMHs in the bilateral centrum semiovale, paraventricular area, cerebellum, and brainstem (Fig. 4I and J).

### Inherited CNS hypomyelinating leukodystrophies

Two patients were diagnosed with 4H syndrome, a condition characterized by CNS hypomyelination, due to compound heterozygous mutations in the *POLR3A* and *POLR3B* genes, respectively. The first patient, a 49‐year‐old woman, presented with a 20‐year history of ataxia and limb weakness. MRI revealed T2‐weighted hyperintensity of the left parietal lobe and bilateral cerebral peduncles. The second patient, a 32‐year‐old woman, experienced a cognitive decline for 25 years and had an epileptic seizure at the age of 24, followed by intermittent dizziness. MRI demonstrated diffuse, symmetric T2 hyperintensity in the cerebral white matter bilaterally and slight brain atrophy (Fig. 4K). Additional examinations revealed hypodontia and myopia, but normal gonadal function in the second patient.

### Other diseases with white matter involvement

In this study, we identified several patients with other genetic disorders presenting with prominent white matter involvement that were not leukodystrophies. Two patients were identified with homozygous or compound heterozygous *ATP7B* mutations, consistent with the diagnosis of Wilson disease. One of these patients presented with dysarthria and dysphagia at 19 years of age and developed parkinsonism over the following 13 years. MRI revealed the presence of scattered symmetrical WMHs in various regions of the brain. The other patient presented with mild postural tremor and progressive dysarthria over a 20‐year period and was diagnosed with symmetrical WMHs in the frontoparietal lobe, external capsule, midbrain, and pons based on brain MRI at 39 years of age (Fig. 4L).

In addition, two patients were diagnosed with PFBC due to *JAM2* and *PDGFB* mutations, respectively. The patient with a homozygous *JAM2* mutation developed ataxia at age 31, while the patient with a heterozygous *PDGFB* mutation developed dizziness and fever at 18 years old. Brain MRI showed symmetrical WMHs in the frontoparietal and temporal lobes, as well as in the periventricular areas. SWI and CT imaging showed symmetric calcifications in the subcortical region, cerebellum, thalami, basal ganglia, and cerebral peduncle.

### Comparison of genetic profiles in different populations

Comparison of genetic profiles among distinct populations was conducted to elucidate the ethnic disparities in adult leukoencephalopathies. Data on other populations were obtained from previous studies.^5^, ^6^, ^8^, ^9^, ^32^, ^33^ In some cases, due to the unavailability of WES/focused exome sequencing or dynamic mutation analysis in certain studies, results were compared separately.

### Comparison of WES/Focused exome sequencing data

**Figure 5:**
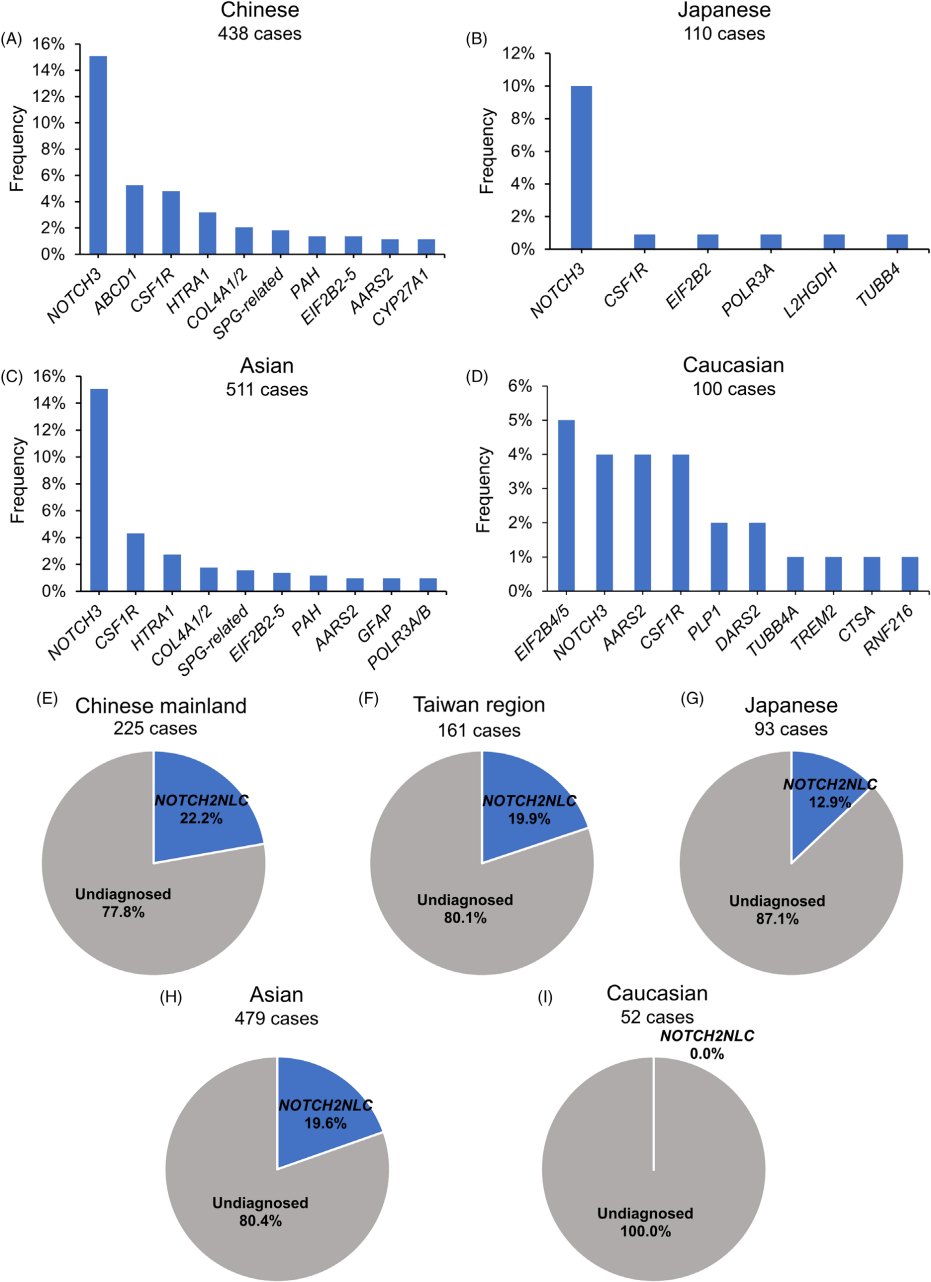
Genetic profiles of adult leukoencephalopathies in different populations. (A–D) Top mutations identified by whole‐exome sequencing (WES) in Chinese (A), Japanese (B), Asian (combination of Chinese and Japanese cohorts) (C), and Caucasian (combination of British and Brazilian cohort) (D). (E–I) The proportion of the *NOTCH2NLC* mutation in WES‐negative leukoencephalopathies in the Chinese mainland, Taiwan region (F), Japanese (G), Asian (combination of Chinese mainland, Taiwanese and Japanese cohorts) (H), and Caucasian (a British cohort) (I).

The prevalence of mutated genes associated with adult leukoencephalopathies in Asians (primarily Chinese and Japanese) and Caucasians (mostly British and Brazilian) was summarized using WES or focused exome sequencing (Fig. 5A–D). In the Chinese cohorts, a genetic diagnosis was established in 47.3% of 438 cases, with *NOTCH3* (15.1%), *ABCD1* (5.3%), *CSF1R* (4.8%), *HTRA1* (3.2%), *COL4A1/2* (2.1%), *SPG‐related genes* (1.8%), *PAH* (1.4%), *EIF2B2–5* (1.4%), *AARS2* (1.1%), and *CYP27A1* (1.1%) being the most frequently identified mutations (Fig. 5A). Similarly, in the Japanese cohort of 110 cases, the genetic diagnosis rate was 14.5%, with *NOTCH3* (10.0%), *CSF1R* (0.9%), *EIF2B2* (0.9%), *POLR3A* (0.9%), *L2HGDH* (0.9%), and *TUBB4* (0.9%) being the most frequent causative genes (Fig. 5B).^6^, ^8^ In the British and Brazilian cohorts of 100 cases, the genetic diagnosis rate was 26.0% due to the exclusion of classical leukodystrophies such as ALD, Krabbe disease, and CTX. *EIF2B4/5* was the most frequently mutated gene (5.0%), followed by *NOTCH3* (4.0%), *AARS2* (4.0%), *CSF1R* (4.0%), *PLP1* (2.0%), *DARS2* (2.0%), *TUBB4A* (1.0%), *TREM2* (1.0%), *CTSA* (1.0%), and *RNF216* (1.0%).^5^ Furthermore, in comparing the genetic spectrum of Asian and Caucasian populations, genes responsible for classical leukodystrophies, including *ABCD1*, *CYP27A1*, *GALC*, *CBS*, and *mDNA*, were excluded in the Asian cohort to minimize bias caused by varying diagnostic criteria. The results indicated that *NOTCH3* was the most frequent causative gene in the Asian cohort, whereas *EIF2B4/5* was the most frequent in the Caucasian cohort (Fig. 5C and D).

### Comparison of dynamic mutation data

Screening for expansions of the GGC repeat region in *NOTCH2NLC* was conducted in cohorts of Chinese mainland, Taiwan region, Japanese, and British individuals (Fig. 5E–I). Among our cohort of 78 patients with adult leukoencephalopathy who were negative for WES, 11 (14.1%) were found to carry an expanded *NOTCH2NLC* allele, with a median repeat size of 104. In a study by Wu et al., 39 (26.5%) out of 147 WES‐negative patients were found to harbor an expanded *NOTCH2NLC* allele, with a median GGC repeat size of 120.^33^ These findings suggest that abnormal expansions of the *NOTCH2NLC* GGC repeat region may contribute to up to 22.2% of unresolved leukoencephalopathies in Chinese mainland populations (Fig. 5E). In a cohort of Taiwan region patients with genetically unsolved non‐vascular leukoencephalopathies, 32 (19.8%) out of 161 were found to have an expanded *NOTCH2NLC* allele, with a median GGC repeat size of 116 (Fig. 5F).^32^ In a Japanese cohort of patients with adult leukoencephalopathy, 12 (12.9%) out of 93 carried an abnormal *NOTCH2NLC* GGC expansion, with a median repeat size of 115 (Fig. 5G).^8^ Taken together, *NOTCH2NLC* GGC expansions account for approximately 19.6% of WES‐unresolved leukoencephalopathy cases in the Asian population (Fig. 5H). In contrast, in a British cohort of 52 patients with adult‐onset leukoencephalopathy without a known genetic diagnosis, none were found to have an expanded *NOTCH2NLC* allele (Fig. 5I).^9^ These results suggest that while *NOTCH2NLC* is an important genetic component of adult leukoencephalopathy in individuals of Asian descent, it may not play a significant role in patients of Caucasian ancestry.

## Discussion

This study conducted a comprehensive genetic investigation to identify the genetic spectrum for adult leukoencephalopathies in a Chinese cohort, and established a relationship between genotypes and clinical phenotypes. The results showed that WES combined with dynamic mutation analysis increased the diagnostic rate to 48.1% in the leukoencephalopathy cohort, with *NOTCH3* and *NOTCH2NLC* being the most commonly mutated genes. The different mutant genotypes were found to have great differences in phenotypes. The comparison of genetic profiles demonstrated a different mutational spectrum for adult leukoencephalopathies in Asians than in Caucasians.

Although WES technology has significantly improved the diagnosis of leukoencephalopathies, it has its limitations, such as difficulty in detecting copy number variations and non‐coding area variations.^34^ Recent long‐read sequencing technology has solved these problems, as evidenced by the discovery of noncoding CGG repeat expansions of *NOTCH2NLC*, *FMR1*, and *NUTM2B‐AS1* in three leukoencephalopathies patients based on long‐read sequencing technology.^7^, ^11^, ^35^, ^36^ This suggests an important role of dynamic mutation in genetic leukoencephalopathies.

In this study, we investigated dynamic mutations in Chinese patients with adult leukoencephalopathies and discovered that 8.5% of patients had *NOTCH2NLC* GGC repeat expansions, but none had *FMR1* and *NUTM2B‐AS1* repeat expansions. Cognitive decline, autonomic dysfunction, limb weakness, headache, and parkinsonism were observed as common manifestations of NIID. Additionally, we observed a stroke‐like episode in one patient, which initially led to a misdiagnosis. Previous studies have identified episodic encephalopathy as a specific manifestation with a strong diagnostic value for NIID. These episodes include sudden disturbance of consciousness, stroke‐like episodes, encephalitis‐like episodes, or psychiatric symptoms, observed in 5.0%–66.7% of NIID patients.^37^ Besides, high‐intensity signal along the corticomedullary junction that affects subcortical U‐fibers on DWI is considered a reliable indicator of NIID.^37^ However, this feature was absent in 18.2% of our NIID patients, indicating insufficient sensitivity. This similar radiological change was also observed in FXTAS and OPML patients, suggesting inadequate specificity of this feature.^7^ Therefore, overemphasizing such high‐intensity DWI signals may result in misdiagnosis or underdiagnosis. Further genetic investigations are warranted for patients exhibiting such features.

The vasculopathy‐related genes were found to be common causes of adult leukoencephalopathies in different ethnic populations, but the mutational hotspot and clinical features had great genetic heterogeneities. Cysteine‐related mutations in *NOTCH3* were confirmed to be the cause of CADASIL, but recent studies have also identified the pathogenicity of cysteine‐sparing *NOTCH3* mutations, such as the p.R75P mutation in Korea and Japan.^38^, ^39^ Some novel cysteine‐sparing mutations in *NOTCH3*, such as p.R90H and p.V237M, were also found in our study. In addition, most of our CADASIL patients presented with ischemic stroke or recurrent TIA and a few suffered from migraine, which was different from Caucasians due to the higher proportion of migraine in Caucasians.^40^

Inherited metabolic disorders are common causes of child leukoencephalopathies,^41^ but they were found not to be rare in our adult leukoencephalopathies cohort. Several metabolic disorders were identified that affected very long chain fatty acid profile (ALD), serum cholestanol/urinary bile alcohols (CTX), specific enzyme activities (PKU and Krabbe disease) and plasma amino acid profile (homocystinuria).^42^, ^43^, ^44^, ^45^, ^46^ The study failed to detect the ARSA mutation that causes metachromatic leukodystrophy, the most frequent diagnosis among childhood leukoencephalopathies, which may be attributed to different genetic profiles.^41^ Inherited metabolic disorders with leukoencephalopathies have an earlier age at onset, a milder phenotype with a slower progression, and common features such as spastic gait, ataxia, cognitive decline, and peripheral neuropathy. Besides, white matter involvement on MRI is usually symmetrical and widely distributed.

This is the first study to compare the genetic profiles of adult leukoencephalopathies in different populations. Our study suggested that the genetic spectrum of adult leukoencephalopathy patients was different between Asians and Caucasians but similar within Asians. *NOTCH3* mutations were classical causes of both Asians and Caucasians. However, *NOTCH2NLC* mutations were the hotspot of Asians instead of Caucasians. The proportion of *NOTCH2NLC* mutations, as well as the number of GGC repeats, is similar in different regions of Asia. We speculate that the Asian population has a founder effect on the *NOTCH2NLC* mutation. The fact that *NOTCH2NLC* GGC expansions appear to be more common in Asian populations compared to Caucasian populations is an important finding, and has implications for clinical practice. Clinicians should consider screening for these expansions in patients with adult leukoencephalopathy of Asian descent who have undergone negative WES testing. It is important to note, however, that *NOTCH2NLC* expansions do not account for all cases of adult leukoencephalopathy in Asian populations. Therefore, further research is needed to identify other genetic causes of this condition in these populations. Additionally, it remains unclear why *NOTCH2NLC* expansions appear to be more prevalent in certain ethnic groups, and future studies should explore this question further.

The study had some limitations. First, in clinical practice, suspected cases of genetic leukoencephalopathies are typically evaluated using a diagnostic algorithm that includes the exclusion of classical leukodystrophies via hematological, biochemical, and metabolic testing.^1^ However, since classical leukodystrophies are also considered genetic leukoencephalopathies, we included these patients in our study and performed genetic testing to obtain a complete genetic profile. Second, it is important to note that different inclusion criteria were used in previously published studies when comparing the genetic profiles of different populations. For example, classical leukodystrophies were excluded in the Caucasian cohort, which makes it inappropriate to directly compare the proportion of different diseases in these studies.^5^ To reduce the bias caused by different diagnostic criteria, we excluded the genes responsible for classical leukodystrophies. Despite our efforts, the preliminary conclusion regarding the genetic profiles of adult leukoencephalopathies in Asian and Caucasian populations should be considered with caution, and further well‐designed case‐control studies will be needed to verify the genetic heterogeneity among different populations.

In contemporary clinical practice, gene diagnoses have become increasingly important due to the critical role they play in the management and treatment of various genetic disorders. This study highlights the significance of genetic testing in diagnosing leukoencephalopathies, which is imperative for conducting systematic research on the management and treatment of these disorders. For instance, hematopoietic stem‐cell transplantation (HSCT) has recently been established as an effective therapeutic strategy for HDLS, ALD, and other leukoencephalopathies.^47^, ^48^, ^49^, ^50^ Additionally, substrate reduction therapy has shown promise in reducing the accumulation of storage material in Krabbe disease.^51^

In summary, this study presents a comprehensive genetic approach to diagnose approximately 50% of Chinese patients with adult leukoencephalopathies. The identification of common pathogenic genes such as *NOTCH3*, *NOTCH2NLC*, *CSF1R*, and *ABCD1* serves to improve clinical recognition and inform genotype–phenotype correlation analysis. Furthermore, the comparison of genetic profiles highlights a different genetic spectrum for adult leukoencephalopathies in Asian and Caucasian populations, emphasizing the need for population‐specific research on the management and treatment of these disorders.

## Author Contributions

JY, CS, and YX conceived the project and designed the study. ML, YW, YY, LL, and XZ participated in the clinical data acquisition and analysis. ML, YW, and YY conducted the experiments and analyzed the data. LL, XZ, and CS participated in the literature search and analysis. ML and JY drafted the original manuscript. All the authors approved the final version.

## Conflict of Interest

The authors report no conflict of interest.

## Supporting information


**Table S1** Known pathogenic/likely pathogenic variants identified in our cohort.

**Table S2** Variants of uncertain significance identified in our cohort.

**Table S3** Clinical features of patients identified with genetic leukoencephalopathies.

**Table S4** Imaging appearance of patients identified with genetic leukoencephalopathies.
