# Supplementary material for: Genetic spectrum and clinical features of adult leukoencephalopathies in a Chinese cohort
Source: Ann Clin Transl Neurol. 2023 May 26;10(7):1119–35. doi: 10.1002/acn3.51794 (PMC10351660; doi:10.1002/acn3.51794)
Supplement: Supplementary file 1 — Table S1 Known pathogenic/likely pathogenic variants identified in our cohort. [file ACN3-10-1119-s004.docx]

**Supplementary Table 1 Known pathogenic/likely pathogenic variants identified in our cohort**

| **Gene** | **Nucleotide change** | **Amino acid change** | **Inheritance pattern** | **ACMG** |
| --- | --- | --- | --- | --- |
| *NOTCH3* | c.328C>T | p.R110C | AD | P (PS4+PM1+PM2+PP1+PP2+PP3) |
| *NOTCH3* | c.397C>T | p.R133C | AD | LP (PS4+PM1+ PM2+ PP2+ PP3+ PP5) |
| *NOTCH3* | c.505C>T | p.R169C | AD | P (PVS1+PS4+PM2+PP2) |
| *NOTCH3* | c.554G>A | p.C185Y | AD | LP (PM1+PM2+PM5+PP3) |
| *NOTCH3* | c.1630C>T | p.R544C | AD | P (PS1+PM1+PM2+PP2+PP3+PP5) |
| *NOTCH3* | c.1819C>T | p.R607C | AD | P (PS4+PM1+PM2+PP2+PP3) |
| *NOTCH3* | c.2951T>G | p.F984C | AD | P (PS1+PM1+PM2+PP1+PP2+PP3+PP4) |
| *NOTCH3* | c.3062A>G | p.Y1021C | AD | LP (PS4+PM2+PP2+PP3) |
| *CSF1R* | c.1765G>A | p.G589R | AD | LP (PS4+PM1+PM2+PP3) |
| *CSF1R* | c.2297T>C | p.M766T | AD | LP (PM1+PM2+PP1+PP3+PP4) |
| *CSF1R* | c.2381T>C | p.I794T | AD | P (PS1+PM1+PM2+PP1+PP3+PP4) |
| *ATP7B* | c.2333G>T | p.R778L | AR | P (PS1+PS4+PM1+PP3) |
| *ATP7B* | c.3316G>A | p.V1106I | AR | LP (PS4+PM2+PP3) |
| *ATP7B* | c.3700delG | - | AR | VUS (PM2) |
| *ABCD1* | c.1166G>A | p.R389H | XR | LP (PS4+PM2+PM5+PP3） |
| *ABCD1* | c.1252C>T | p.R418W | XR | P (PS4+PM1+PM2+PP3+PP4) |
| *ABCD1* | c.1415_1416del | p.Q472fs | XR | P (PVS1+PM2+PP5) |
| *ABCD1* | c.1661G>A | p.R554H | XR | P (PS4+PM1+PM2+PP3+PP4) |
| *CYP27A1* | c.1016C>T | p.T339M | AR | LP (PS4+PM1+PM2+PP3) |
| *CYP27A1* | c.1263+1G>A | - | AR | P (PVS1+PS4+PM2) |
| *COL4A1* | c.*32G>A | - | AD | P (PS3+PM2+PP1+PP3+PP4) |
| *HTRA1* | c.824C>T | p.P275L | AR | LP (PS4+PM1+PM2+PP3) |
| *GFAP* | c.1246C>T | p.R416W | AD | P (PS3+PS4+PM2+PP3) |
| *PAH* | c.440C>T | p.P147L | AR | LP (PS1+PM1+PM2+PP3) |
| *PAH* | c.511G>A | p.G171R | AR | LP (PM2+PM3+PP3+PP4) |
| *PAH* | c.1197A>T | p.V399V | AR | P (PS3+PS4+PM2) |
| *POLR3A* | c.1771-7C>G | - | AR | P (PS3+PM2+PM3+PP1) |
| *POLR3A* | c.3718G>A | p.G1240S | AR | LP (PM1+PM2+PM3+PP3) |
| *DARS2* | c.228-16C>A | - | AR | P (PS3+PM2+PM3) |
| *DARS2* | c.787C>T | p.R263* | AR | P (PVS1+PM2+PM3) |
| *CBS* | c.374G>A | p.R125Q | AR | LP (PS3+PS4+PM2+PM3) |
| *CBS* | c.1330G>A | p.D444N | AR | P (PVS1+PS3+PS4+PM2+PM3) |
| *AARS2* | c.452T>C | p.M151T | AR | LP (PM1+PM2+PM3+PP3+PP4) |
| *DPYD* | c.2303C>A | p.T768L | AR | LP (PS3+PM1+PM3+PP3) |
| *GALC* | c.1901T>C | p.L634S | AR | LP (PS4+PM2+PM5+PP3) |

Abbreviation: AD, autosomal dominant; AR, autosomal recessive; XR, X-linked recessive; ACMG, American College of Medical Genetics and Genomics; P, pathogenic; LP, likely pathogenic; VUS, variants of uncertain significance.
