# Supplementary material for: Genetic spectrum and clinical features of adult leukoencephalopathies in a Chinese cohort
Source: Ann Clin Transl Neurol. 2023 May 26;10(7):1119–35. doi: 10.1002/acn3.51794 (PMC10351660; doi:10.1002/acn3.51794)
Supplement: Supplementary file 2 — Table S2 Variants of uncertain significance identified in our cohort. [file ACN3-10-1119-s003.docx]

**Supplementary Table 2 Variants of uncertain significance identified in our cohort**

| **Gene** | **Nucleotide change** | **Amino acid change** | **Inheritance pattern** | **Genotype** | **gnomAD** | **ExAC** | **SIFT** | **Polyphen-2** | **CADD** | **ACMG** | **Novelty** |
| --- | --- | --- | --- | --- | --- | --- | --- | --- | --- | --- | --- |
| *NOTCH3* | c.6074A>G | p.D2025G | AD | Het | - | - | D | Pos_D | D | VUS (PM2,PP3,PP4) | N |
| *NOTCH3* | c.3901G>A | p.G1301S | AD | Het | 9.83E-06 | - | D | Pro_D | D | VUS (PM2,PP3,PP4) | N |
| *NOTCH3* | c.5548G>A | p.A1850T | AD | Het | 1.63E-05 | 1.66E-05 | D | Pro_D | D | VUS (PM2,PP3,PP4) | N |
| *NOTCH3* | c.3785G>A | p.R262H | AD | Het | - | - | D | B | D | VUS (PM2,PP2,PP4,BP4) | N |
| *HTRA1* | c.1004T>C | p.L335P | AD | Het | - | - | D | Pro_D | D | VUS (PM2,PP3,PP4) | N |
| *HTRA1* | c.523G>A | p.V175M | AD | Het | 4.06E-06 | 8.24E-06 | D | Pro_D | D | VUS (PM2,PP3,PP4) | K |
| *HTRA1* | c.34-35insCT | p.L12fs | AR | Het | - | - | NA | NA | NA | VUS (PM2,PP4) | N |
| *HTRA1* | c.472+6C>A | - | AR | Het | - | - | NA | NA | NA | VUS (PM2,PP4) | N |
| *COL4A1* | c.903+39(IVS16)G>C | - | AD | Het | - | - | NA | NA | NA | VUS (PM2,PP4) | N |
| *COL4A1* | c.3589G>T | p.A1197S | AD | Het | - | - | T | B | D | VUS (PM2,PP4) | N |
| *COL4A1* | c.2628T>A | p.G876G | AD | Het | 4.06E-06 | - | NA | NA | NA | VUS (PM2) | N |
| *APP* | c.2059A>C | p.K687Q | AD | Het | - | - | D | Pro_D | D | VUS (PM2,PP3) | N |
| *ABCD1* | c.415_417delCTC | p.L139del | XR | Hemi | - | - | NA | NA | NA | VUS (PM2,PP4) | N |
| *ABCD1* | c.2065c>T | p.R689C | XR | Hemi | 7.12E-05 | 6.96E-05 | D | Pro_D | D | VUS (PM2,PP3,PP4) | N |
| *ABCD1* | c.276dupG | p.L93fs | XR | Hemi | - | - | NA | NA | NA | VUS (PM2,PP4) | N |
| *ATN1* | c.3358+1G>A | Splicing | AD | Het | - | - | NA | NA | NA | VUS (PM2,PP4) | N |
| *CSF1R* | c.2416G>A | p.D806N | AD | Het | 8.12E-06 | 8.24E-06 | D | Pro_D | D | VUS (PM2,PP3,PP4) | N |
| *GFAP* | c.1257G>C | p.E419D | AD | Het | - | - | NA | NA | T | VUS (PM2,PP4) | N |

Abbreviation: gnomAD, Genome Aggregation Database; ExAC, Exome Aggregation Consortium; ACMG, American College of Medical Genetics and Genomics; AD, autosomal dominant; AR, autosomal recessive; XR, X-linked recessive; Het, heterozygous; D, damaging; NA, not available; T, tolerant; Pos_D, possible damaging; Pro_D, probably damaging; B, benign; VUS, variants of uncertain significance; N, novel; K, known.
