# Supplementary material for: Genetic spectrum and clinical features of adult leukoencephalopathies in a Chinese cohort
Source: Ann Clin Transl Neurol. 2023 May 26;10(7):1119–35. doi: 10.1002/acn3.51794 (PMC10351660; doi:10.1002/acn3.51794)
Supplement: Supplementary file 3 — Table S3 Clinical features of patients identified with genetic leukoencephalopathies. [file ACN3-10-1119-s001.docx]

**Supplementary Table 3 Clinical features of patients identified with genetic leukoencephalopathies**

| **Proband number** | **Sex** | **Age** | **Age at onset** | **Familial history** | **Initial symptoms** | **Additional symptoms** | **MMSE** | **Gene** | **Variants/**  **Repeats** | **Diagnosis** |
| --- | --- | --- | --- | --- | --- | --- | --- | --- | --- | --- |
| 1 | M | 33 | 33 | + | Ischemic stroke | - | / | *NOTCH3* | c.554G>A | CADASIL |
| 2 | M | 58 | 52 | + | Cognitive decline | Ataxia, ischemic stroke | 9 | *NOTCH3* | c.269G>A | CADASIL |
| 3 | F | 43 | 43 | + | Ischemic stroke | Ataxia | 22 | *NOTCH3* | c.985T>C | CADASIL |
| 4 | M | 22 | 22 | + | Ischemic stroke | - | / | *NOTCH3* | c.2951T>G | CADASIL |
| 5 | F | 40 | 40 | + | Migraine | - | 24 | *NOTCH3* | c.3172G>T | CADASIL |
| 6 | F | 54 | 54 | + | Migraine | TIA, cognitive decline | 19 | *NOTHC3* | c.397C>T | CADASIL |
| 7 | F | 56 | 49 | + | Cognitive decline | Sleep disorder | 17 | *NOTHC3* | c.1819C>T | CADASIL |
| 8 | F | 48 | 43 | + | Migraine | TIA, cognitive decline | / | *NOTCH3* | c.3062A>G | CADASIL |
| 9 | M | 34 | 34 | + | Ischemic stroke | - | / | *NOTCH3* | c.328C>T | CADASIL |
| 10 | M | 31 | 21 | - | Parkinsonism | Ataxia | / | *NOTCH3* | c.1649C>T | CADASIL |
| 11 | F | 72 | 70 | + | Recurrent TIA | Behavioural change, ischemic stroke | 2 | *NOTCH3* | c.505C>T | CADASIL |
| 12 | M | 58 | 54 | + | Ischemic stroke | Hemorrhagic stroke, dizziness | / | *NOTCH3* | c.5467G>A | CADASIL |
| 13 | M | 43 | 41 | + | Migraine | Dizziness, cognitive decline, hearing loss | / | *NOTCH3* | c.709G>A | CADASIL |
| 14 | M | 48 | 45 | + | Recurrent TIA | Dizziness | / | *NOTCH3* | c.1063T>C | CADASIL |
| 15 | M | 21 | 20 | + | Dizziness | - | / | *NOTCH3* | c.1063T>C | CADASIL |
| 16 | M | 54 | 54 | + | Ischemic stroke | Dizziness, ataxia | / | *NOTCH3* | c.1630C>T | CADASIL |
| 17 | M | 47 | 45 | + | Ischemic stroke | Depression, cognitive decline | 21 | *HTRA1* | c.62C>A | CADASIL2 |
| 18 | F | 55 | 49 | + | Ischemic stroke | Personality change, cognitive decline | 19 | *HTRA1* | c.971A>G | CADASIL2 |
| 19 | M | 54 | 53 | - | Spastic gait | Low back pain, cognitive decline, parkinsonism | 22 | *HTRA1* | c.824C>T;  c.176G>C | CARASIL |
| 20 | M | 42 | 40 | + | Ischemic stroke | Ataxia | / | *COL4A1* | c.*32G>A | BSVD1 |
| 21 | M | 37 | 36 | + | Ischemic stroke | Ataxia, limited eye movement | / | *COL4A1* | c.*32G>A | BSVD1 |
| 22 | F | 43 | 42 | - | Parkinsonism | Ataxia, cognitive decline | 18 | *COL4A1* | E16-17 del | BSVD1 |
| 23 | M | 49 | 38 | - | Peripheral neuropathy | Rigidity, spastic gait | 28 | *ABCD1* | c.1166G>A | ALD |
| 24 | M | 47 | 40 | + | Peripheral neuropathy | Rigidity, spastic gait | 29 | *ABCD1* | c.1415_1416del | ALD |
| 25 | M | 48 | 47 | - | Spastic gait | Peripheral neuropathy, behavioural change, cognitive decline, autonomic dysfunction | 22 | *ABCD1* | c.1661G>A | ALD |
| 26 | M | 32 | 31 | - | Dysarthria | Dysphagia, cognitive decline, tremor, autonomic dysfunction | / | *ABCD1* | c.1252C>T | ALD |
| 27 | M | 18 | 8 | - | Muscle atrophy, pescavu | Peripheral neuropathy, bradykinesia, | / | *CYP27A1* | c.1016C>T;  c.1263+1G>A | CTX |
| 28 | F | 25 | 19 | - | Epilepsy | Ataxia, personality change, cognitive decline, cataract | 19 | *CYP27A1* | c.1004C>T;  c.1004C>T | CTX |
| 29 | F | 34 | 28 | - | Ataxia | Cognitive decline, dysarthria, dysphagia, cataract | 3 | *CYP27A1* | c.1263+1G>A;  c.1263+1G>A | CTX |
| 30 | M | 18 | 13 | - | Headache | Delusional disorder | 18 | *PAH* | c.440C>T;  c.1197A>T | PKU |
| 31 | M | 21 | 21 | - | Cognitive decline | Ataxia, personality change | 10 | *PAH* | c.511G>A;  c.1197A>T | PKU |
| 32 | M | 53 | 41 | - | Limb numbness, weakness | - | / | *CBS* | c.1330G>A;  c.374G>A | Homocystinurias |
| 33 | M | 33 | 33 | - | Cognitive decline | Blurred vision | 20 | *DPYD* | c.751C>T;  c.2303C>A | DPD Deficiency |
| 34 | M | 27 | 17 | - | Epilepsy | Diplopia, blurred vision | / | *GALC* | c.1901T>C;  c.1901T>C | Krabbe Disease |
| 35 | F | 50 | 49 | - | Cognitive decline | Parkinsonism, ataxia, sleep disorder | 19 | *CSF1R* | c.1949T>C | HDLS |
| 36 | F | 50 | 49 | + | Parkinsonism | Depression, cognitive decline | 17 | *CSF1R* | c.2570C>T | HDLS |
| 37 | M | 38 | 36 | + | Dysarthria | Depression, personality change, peripheral neuropathy, autonomic dysfunction | 18 | *CSF1R* | c.2381T>C | HDLS |
| 38 | F | 30 | 29 | + | Dizziness | Dysarthria, ataxia, autonomic dysfunction | / | *CSF1R* | c.2381T>C | HDLS |
| 39 | F | 44 | 42 | + | Dysarthria | Peripheral neuropathy, parkinsonism, ataxia, depression, cognitive decline | 13 | *CSF1R* | c.1736G>A | HDLS |
| 40 | M | 35 | 35 | - | Dysarthria | Peripheral neuropathy, cognitive decline | 19 | *CSF1R* | c.1765G>A | HDLS |
| 41 | M | 36 | 35 | + | Ataxia | Cognitive decline, dizziness | / | *CSF1R* | c.2297T>C | HDLS |
| 42 | F | 38 | 34 | + | Limb weakness | Bradykinesia, ataxia | / | *GFAP* | c.1246C>T | AxD |
| 43 | M | 67 | 64 | + | Parkinsonism | Autonomic dysfunction | 27 | *GFAP* | c.263G>A | AxD |
| 44 | M | 21 | 19 | - | Ataxia | Headache, tremor | / | *AARS2* | c.452T>C;  c.2557C>T | LKENP |
| 45 | M | 18 | 18 | - | Ataxia | Peripheral neuropathy | / | *DARS2* | c.787C>T;  c.228-16C>A | LBSL |
| 46 | F | 49 | 29 | - | Ataxia | Muscle weakness | / | *POLR3A* | c.1771-7C>G;  c.3718G>A | 4H syndrome |
| 47 | F | 32 | 7 | - | Cognitive decline | Epilepsy, ataxia | 13 | *POLR3B* | c.361C>T;  c.2698C>T | 4H syndrome |
| 48 | F | 32 | 19 | - | Dysarthria, dysphagia | Parkinsonism | / | *ATP7B* | c.2333G>T;  c.2333G>T | WD |
| 49 | M | 39 | 37 | - | Postural tremor | Dysarthria | / | *ATP7B* | c.3316G>A;  c.3700delG | WD |
| 50 | M | 32 | 31 | - | Ataxia | - | / | *JAM2* | c.460C>T;  c.460C>T | PFBC |
| 51 | M | 18 | 18 | - | Dizziness | Fever | / | *PDGFB* | c.148G>T | PFBC |
| 52 | F | 76 | 76 | - | Cognitive decline | Limb weakness, loss of consciousness, behavioural change, autonomic dysfunction | 2 | *NOTCH2NLC* | 104 | NIID |
| 53 | F | 52 | 52 | - | Cognitive decline | Headache, limb numbness, autonomic dysfunction | 28 | *NOTCH2NLC* | 65 | NIID |
| 54 | M | 48 | 48 | + | Headache | Sleep disorder, autonomic dysfunction | 28 | *NOTCH2NLC* | 133 | NIID |
| 55 | F | 68 | 67 | - | Limb weakness | Cognitive decline, autonomic dysfunction | 7 | *NOTCH2NLC* | 176 | NIID |
| 56 | F | 57 | 47 | - | Headache | Cognitive decline, hearing loss | 29 | *NOTCH2NLC* | 111 | NIID |
| 57 | M | 48 | 47 | - | Limb numbness | Dizziness, autonomic dysfunction | 26 | *NOTCH2NLC* | 126 | NIID |
| 58 | F | 70 | 70 | - | Limb weakness | Cognitive decline, parkinsonism, ataxia | 0 | *NOTCH2NLC* | 96 | NIID |
| 59 | M | 52 | 46 | - | Cognitive decline | Peripheral neuropathy, ataxia | 20 | *NOTCH2NLC* | 114 | NIID |
| 60 | M | 63 | 62 | + | Cognitive decline | Loss of consciousness | 8 | *NOTCH2NLC* | 93 | NIID |
| 61 | F | 62 | 57 | - | Limb weakness | Parkinsonism, depression | 25 | *NOTCH2NLC* | 85 | NIID |
| 62 | M | 63 | 58 | + | TIA | Cognitive decline, parkinsonism, headache | 10 | *NOTCH2NLC* | 99 | NIID |

Abbreviation: M, male; F, female; “+”, positive; “-”, negative; MMSE, Mini-mental State Examination; TIA, transient ischemic attack; CADASIL, cerebral autosomal dominant arteriopathy with subcortical infarcts and leukoencephalopathy; CARASIL, cerebral autosomal recessive arteriopathy with subcortical infarcts and leukoencephalopathy; BSVD1, brain small vessel disease with or without ocular anomalies; ALD, X-linked adrenoleukodystrophy; CTX, Cerebrotendinous Xanthomatosis; PKU, phenylketonuria; DPD, dihydropyrimidine dehydrogenase; HDLS, hereditary diffuse leukodystrophy with axonal spheroids; AxD, Alexander disease; LKENP, progressive leukodystrophy with ovarian failure; LBSL, leukoencephalopathy with brainstem and spinal cord involvement and lactate elevation; WD, Wilson disease; PFBC, primary familial brain calcification; NIID, neuronal intranuclear inclusion disease.
