# Supplementary material for: Genetic spectrum and clinical features of adult leukoencephalopathies in a Chinese cohort
Source: Ann Clin Transl Neurol. 2023 May 26;10(7):1119–35. doi: 10.1002/acn3.51794 (PMC10351660; doi:10.1002/acn3.51794)
Supplement: Supplementary file 4 — Table S4 Imaging appearance of patients identified with genetic leukoencephalopathies. [file ACN3-10-1119-s002.docx]

**Supplementary Table 4 Imaging appearance of patients identified with genetic leukoencephalopathies**

|  | Cerebral white matter hyperintensity on T2/FLAIR | | | | | | | | | Associated Features | | | | |
| --- | --- | --- | --- | --- | --- | --- | --- | --- | --- | --- | --- | --- | --- | --- |
| Proband number | Frontoparietal | Temporal | Occipital | Periventricular | Subcortical U Fibers | External capsule | Corpus callosum | Brainstem | Cerebellum | DWI abnormality | Calcifications | Infarcts | Microbleeds | Contrast enhancement |
| 1 | + | + | - | + | - | + | - | - | - | + | - | + | - | NA |
| 2 | + | + | - | + | - | - | - | - | + | + | - | + | + | NA |
| 3 | + | + | - | + | - | + | + | - | - | + | - | + | - | NA |
| 4 | + | + | - | + | - | - | - | - | - | + | - | + | - | NA |
| 5 | + | + | - | + | - | + | - | - | - | - | + | - | - | - |
| 6 | + | + | - | + | - | + | - | - | - | + | - | + | - | NA |
| 7 | + | + | + | + | - | + | + | + | + | + | - | - | - | NA |
| 8 | + | + | + | + | - | + | + | - | - | + | - | + | - | - |
| 9 | + | + | + | + | - | + | - | - | - | + | - | + | + | - |
| 10 | + | + | - | + | - | - | - | - | - | - | - | - | - | NA |
| 11 | + | + | - | + | - | + | + | + | - | + | - | + | - | NA |
| 12 | + | + | - | + | - | - | - | - | - | + | + | - | - | NA |
| 13 | + | + | + | + | - | + | + | + | + | - | - | - | - | NA |
| 14 | + | + | - | + | - | + | - | - | - | - | + | - | - | NA |
| 15 | + | - | - | + | - | - | - | - | - | - | - | - | - | NA |
| 16 | + | + | - | + | - | + | + | + | - | - | + | + | + | NA |
| 17 | + | - | - | + | - | - | - | + | + | + | - | + | - | NA |
| 18 | + | - | - | + | - | + | + | + | - | + | - | + | - | NA |
| 19 | + | + | - | + | - | + | + | + | - | - | - | + | + | NA |
| 20 | + | + | + | + | - | - | + | + | + | + | - | + | - | NA |
| 21 | + | - | - | + | - | - | - | + | - | - | - | + | + | - |
| 22 | + | - | - | + | - | - | - | - | - | - | + | + | + | NA |
| 23 | + | - | - | + | - | - | - | - | - | - | + | - | - | NA |
| 24 | + | - | - | + | - | - | - | - | - | + | - | - | - | NA |
| 25 | + | + | + | + | + | + | + | + | - | + | - | - | - | + |
| 26 | + | - | - | + | - | - | + | + | + | + | - | - | - | - |
| 27 | - | - | - | + | - | - | - | - | - | - | - | - | - | NA |
| 28 | - | - | - | + | - | - | - | - | - | - | - | - | - | NA |
| 29 | - | - | - | + | - | - | - | + | + | + | - | - | - | NA |
| 30 | - | - | - | + | - | - | - | - | - | - | - | - | - | NA |
| 31 | + | + | + | + | + | - | + | - | - | + | - | - | - | - |
| 32 | + | - | - | + | - | + | + | + | - | - | - | - | - | NA |
| 33 | + | - | - | - | + | + | + | + | - | + | - | - | - | NA |
| 34 | + | - | - | + | - | - | - | - | - | + | + | - | - | NA |
| 35 | + | + | - | + | - | - | - | - | - | + | + | - | - | - |
| 36 | + | - | - | + | - | - | - | - | - | + | - | + | - | NA |
| 37 | - | - | - | + | - | - | - | - | - | + | - | - | - | - |
| 38 | + | - | - | - | - | - | - | - | - | - | - | - | - | NA |
| 39 | + | + | + | + | + | - | + | + | - | + | - | - | - | NA |
| 40 | + | + | - | + | - | - | + | - | - | + | + | - | - | NA |
| 41 | + | - | + | + | - | - | - | - | - | + | - | - | - | NA |
| 42 | + | - | - | + | - | - | + | + | - | + | - | - | - | NA |
| 43 | + | + | - | + | - | - | - | - | - | + | - | - | - | NA |
| 44 | + | - | - | + | - | - | + | + | - | + | - | - | - | + |
| 45 | + | + | - | + | - | - | + | - | - | + | + | - | - | NA |
| 46 | + | - | - | + | - | - | - | + | - | - | - | - | - | - |
| 47 | + | - | - | + | - | - | - | - | - | - | + | - | - | - |
| 48 | + | - | + | - | - | - | - | - | - | + | - | - | - | NA |
| 49 | + | + | + | + | - | + | + | - | - | + | - | - | - | NA |
| 50 | + | + | - | + | - | - | - | + | + | + | - | - | - | NA |
| 51 | + | - | - | + | - | - | + | - | - | + | - | - | - | NA |
| 52 | + | - | - | + | + | - | - | + | - | + | - | - | - | NA |
| 53 | + | - | - | + | + | - | - | + | - | - | - | - | - | - |
| 54 | + | - | - | + | + | - | + | - | - | + | + | - | + | - |
| 55 | + | + | + | + | + | - | + | - | - | + | + | - | + | NA |
| 56 | + | + | - | + | + | + | - | - | - | + | - | - | - | - |
| 57 | + | - | - | + | + | + | + | - | - | + | - | - | - | NA |
| 58 | + | + | + | + | + | - | + | - | + | + | - | - | - | NA |
| 59 | + | - | - | + | + | - | - | - | - | + | - | + | - | NA |
| 60 | + | - | - | + | + | + | + | + | - | + | + | + | + | NA |
| 61 | + | - | - | + | + | - | + | - | - | + | - | - | - | NA |
| 62 | + | - | - | + | - | - | - | - | - | - | + | - | - | - |

Abbreviation: FLAIR, fluid-attenuated inversion recovery; DWI, diffusion-weighted imaging; “+”, positive; “-”, negative; NA, not available.
